# Assessment of left ventricular mass and volumes by three-dimensional echocardiography in patients with or without wall motion abnormalities: comparison against cine magnetic resonance imaging

**DOI:** 10.1136/hrt.2007.123711

**Published:** 2007-11-01

**Authors:** A-C Pouleur, J-B le Polain de Waroux, A Pasquet, B L Gerber, O Gérard, P Allain, J-L J Vanoverschelde

**Affiliations:** 1 Division of Cardiology, Department of Cardiovascular Diseases, Cliniques Universitaires St Luc, Université Catholique de Louvain, Brussels, Belgium; 2 Philips Medical Systems Research, Paris, France

## Abstract

**Aim:**

To evaluate if three-dimensional echocardiography (3-DE) is as accurate and reproducible as cine magnetic resonance imaging (cMR) in estimating left ventricular (LV) parameters in patients with and without wall motion abnormalities (WMA).

**Methods:**

83 patients (33 with WMA) underwent 3-DE and cMR. 3-DE datasets were analysed using a semi-automatic contour detection algorithm. The accuracy of 3-DE was tested against cMR in the two groups of patients. All measurements were made twice by two different observers.

**Results:**

LV mass by 3-DE was similar to that obtained by cMR (149 (SD 42) g vs 148 (45) g, p = 0.67), with small bias (1 (28) g) and excellent interobserver agreement (−2 (31) g vs 4 (26) g). The two measurements were also highly correlated (r = 0.94), irrespective of WMA. End-diastolic and end-systolic LV volumes and ejection fraction by 3-DE and cMR were highly correlated (r = 0.97, 0.98, 0.94, respectively). Yet, 3-DE underestimated cMR end-diastolic volumes (167 (68) ml vs 187 (70) ml, p<0.001) and end-systolic volumes (88 (56) ml vs 101 (65) ml, p<0.001), but yielded similar ejection fractions (50% (14%) vs 50% (16%), p = 0.23).

**Conclusion:**

3-DE permits accurate determination of LV mass and volumes irrespective of the presence or absence of WMA. LV parameters obtained by 3-DE are also as reproducible as those obtained by cMR. This suggests that 3-DE can be used to follow up patients with LV hypertrophy and/or remodelling.

Left ventricular (LV) volumes, ejection fraction (EF) and mass are important prognostic factors in patients with cardiac diseases and are therefore frequently requested for serial testing.^1^^–^3 Because of its widespread availability, two-dimensional echocardiography (2-DE) is usually the first choice non-invasive imaging method for obtaining these measurements in daily clinical practice.^4^ ^5^ Yet, several studies have shown that one-dimensional M-mode (1-DE) and 2-DE had limited test-retest reproducibility and that their accuracy was impacted by many factors including image quality, foreshortening of the LV cavity and geometric assumptions regarding LV volumes calculation. Because it provides superior image quality, is intrinsically three-dimensional and does not require any geometric assumption for volume calculation, cardiac magnetic resonance (cMR) has progressively become the reference method for assessing LV volumes, EF and mass, especially in clinical trials.^6^ ^7^ However, its cost and availability remain problematic for routine clinical evaluation.

Three-dimensional echocardiography (3-DE) is a promising new method for assessing LV volumes and EF in patients with structural heart disease. When used in combination with computerised endocardial contour tracking algorithms, it indeed permits faster, more accurate and less operator-dependent quantitation of LV volumes and function than 1-DE or 2-DE.^8^^–^11 Preliminary data also suggest that it can be used to measure LV mass.^12^^–^16 So far, however, this requires both the endocardial and the epicardial contours to be traced manually on 3D-derived orthogonal 2-D cross-sections and has only been evaluated in a limited number of patients, most of whom had a normal LV geometry.

We have recently developed a computerised semi-automated volumetric contour tracking algorithm that enables both the endocardial and the epicardial contours to be detected with minimal interaction by the investigator. In this context, the aims of the present study were: (1) to validate this new border detection algorithm for quantitation of LV mass by using cMR as the reference method; (2) to assess the variability of 3-DE estimates of LV mass in patients with or without wall motion abnormalities (WMA); and (3) to evaluate the day-to-day variability of LV mass measurements obtained by 3-DE in comparison with those obtained by cMR.

## METHODS

### Study population

The study population consisted of 83 consecutive subjects (67 men, mean age 54 (SD 19) years, range 7–85 years) who underwent cMR and 3-DE in random order on the same day. There were 20 volunteers (15 men, mean age 29 (13) years, range 7–50 years) and 63 patients with heart disease (52 men, mean age 62 (13) years, range 37–85 years), of whom 20 had aortic valve disease, 10 severe mitral regurgitation and 33 a previous myocardial infarction. All subjects were in sinus rhythm. Patients with haemodynamic instability, constant arrhythmia (atrial fibrillation or more than five premature beats per minute) or any contraindication to cMR (ferrometallic cerebral aneurysm clips, pacemaker or implantable defibrillator, or severe claustrophobia) were not considered for inclusion. In five volunteers, 3-DE and cMR were repeated twice, on two different days, to assess test-retest reproducibility. Ten volunteers and 11 patients with previous myocardial infarction also had 3-DE with and without LV opacification using the commercially available ultrasound contrast agent Sonoview (Bracco, Geneva, Switzerland). The study protocol was approved by the local ethics committee and all patients gave informed consent before inclusion into the study.

### Two-dimensional echocardiography

A complete M-mode, two-dimensional and Doppler examination was performed using a Sonos 7500 system or IE33 (Philips Medical Systems, Andover, MA, USA) with harmonic imaging. Images were acquired from standard parasternal and apical views, with short breath-holds if needed. The presence or absence of regional wall motion abnormalities (WMA) was assessed by using the 16-segments scoring model proposed by the American Society of Echocardiography (1 =  normal wall motion, 2 =  hypokinesia, 3 =  akinesia). LV mass was derived from M-mode echocardiography as previously described.^17^

### Three-dimensional echocardiography

Three-dimensional echocardiography was performed using a Sonos 7500 or a IE33 echocardiographic systems (Philips Medical Systems, Andover, MA, USA) equipped with a dedicated broadband, wide angle, matrix array transducer. Images were acquired from the apical window, with the patient in the left lateral decubitus position. Care was taken to include the entire LV cavity within the pyramidal scan volume. 3-DE datasets were acquired using a wide-angled acquisition (93×80°) mode in which four wedge-shaped subvolumes (93°×20° each) are obtained over four different cardiac cycles during a short breath-hold. Acquisition was triggered to the R wave of every other cardiac cycle to allow time for storage of each subvolume, resulting in a total acquisition time of eight heart beats.

### Cardiac magnetic resonance imaging

cMR imaging was performed on a 1.5 T magnet (Intera CV, Philips Medical Systems, Best, The Netherlands) using a five-element cardiac synergy coil for signal reception. After localisation of the heart using three-plane and oblique survey images, 10–12 contiguous short-axis cine images were prescribed to cover the entire left ventricle from base to apex. Images were acquired using a multislice cine vectocardiographic (VCG) gated balanced fast-field echo sequence with SENSE during serial breath-holds. Twenty cine phases were acquired using retrospective gating with a temporal resolution varying between 25–50 ms. Slice thickness was 8 mm and slice spacing was 2 mm. Imaging parameters were: repetition time 3.1 ms, echo time 1.6 ms, flip angle 60°, field of view 38 cm, image matrix of 160×128 pixels, SENSE acceleration factor 1.6, 12–20 lines acquired per phase.

### Data analysis

Images were transferred to dedicated workstations (QLab and View Forum R4.1 for analysis of 3-DE and cMR images respectively, all Philips Medical systems). Patient information was removed. In all patients, measurements were performed in duplicate by two blinded observers, who were both familiar with 3-DE and cMR. To avoid recalling the patient’s images, the blinded readers analysed 3-DE and cMR images on separate days. To assess intra-observer variability, in a randomly selected subgroup of 45 patients, measurements were repeated by one of the readers, one month after the first reading.

#### Three-dimensional echocardiography

**Figure 1:**
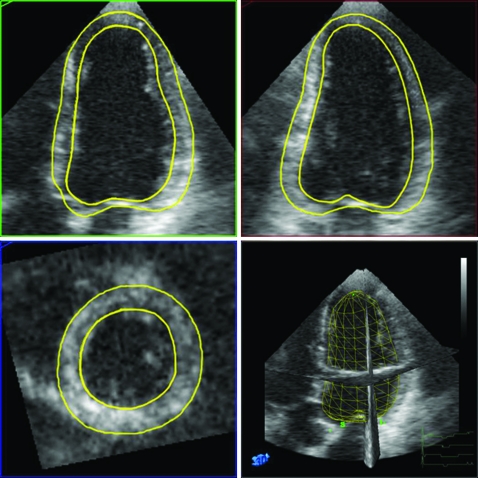
End-diastolic, anatomically correct apical four-chamber and two-chamber views (upper left and right, respectively) and short-axis view (bottom left) selected off-line from a 3-DE dataset obtained in the same subject and resulting 3D-shell (bottom right). Superimposed semi-automatically detected endocardial and epicardial boundaries were used to calculate LV mass.

Image quality of 3-DE images was graded semi-quantitatively by both readers on a five-point scale as 1  =  non-analysable, 2  =  fair, 3  =  moderate, 4  =  good and 5  =  excellent. Measurements of LV volumes, mass and EF were performed off-line. The 3-DE datasets were analysed with a prototype version of 3DQ-QLab software that allows for semi-automatic detection of endocardial and epicardial borders. Briefly, the 3D volume dataset is first displayed in three different cross-sections that can be modified interactively. For the detection algorithm to work properly^18^ the anatomically correct four-chamber and two-chamber views need to be displayed simultaneously. Markers are then placed onto the mitral annulus and the apex, both in end-diastole and end-systole. Using these markers, the software program first creates truncated ellipsoid end-diastolic and end-systolic 3D meshes of the LV. The vertices of these meshes are then automatically attracted to the image borders (3D gradient of image intensity) while respecting prespecified surface smoothing constraints (in order to minimise local curvature on the surface). The end-diastolic and the end-systolic 3D meshes deform until equilibrium is reached between closeness to the borders and minimal curvature. If needed, manual adjustments can be applied. The volumes of the obtained 3D- end-diastolic and end-systolic LV meshes include the papillary muscles into the LV cavity and correspond to the LV end-diastolic and end-systolic LV volumes. The end-diastolic epicardial contours are next automatically estimated by adding a fixed myocardial wall thickness of 8.8 mm to the endocardial mesh.^19^ Manual corrections can then be applied, if needed. These epicardial contours are used to calculate the myocardial volume by subtracting the end-diastolic endocardial volume from the end-diastolic epicardial volume. The difference between these two volumes is then multiplied by the specific mass of myocardial tissue (1.05 g/ml) to obtain LV mass (fig 1).

#### Cine magnetic resonance imaging

cMR loops were analysed off-line with a commercial software (View Forum R4.1). In every end-diastolic short-axis slice, endocardial and epicardial contours were manually traced to calculate LV volumes, mass and EF, while including papillary muscles in the LV cavity.

### Statistical analysis

Statistical analysis was performed using SPSS version 11.5. Values are reported as mean (plus or minus 1 SD). The average of duplicate measurements was used for statistical analysis. Linear regression analysis and Bland-Altman plots with assessment of systematic bias and 95% confidence intervals, as well as two-way mixed intraclass correlation coefficient (ICC) were used to compare LV mass between 1-DE, 3-DE and cMR.^20^ Systematic differences in measurements between these methods were assessed using the Student test. Inter-observer reliability for measurement of LV volumes and mass for each technique was assessed using two-way random, single measure ICC. Intra-observer and intra-subject reliability was assessed using one-way random two measures ICC. All tests were two-sided and a p value <0.05 was considered as statistically significant.

## RESULTS

### Patient characteristics/study protocol

The characteristics of the study population are summarised in table 1. All patients successfully completed 3-DE and cMR. The duration of a complete 3-DE examination was shorter (5 minutes on average, including patient preparation) than that of the cMR examination (25 minutes on average, including patient preparation and acquisition of scout images, reference scan for SENSE and cine short-axis slices). By contrast, the time needed to complete the analysis (LV volumes and mass) tended to be longer with 3-DE (434 (162) seconds, including 25 (7) seconds for automated contour tracking and 408 (160) seconds for manual correction) than for cMR (344 (162) seconds).

**Table 1:** Clinical and echocardiographic characteristics

		All patients	Controls	WMA group
**Clinical characteristics**				
No of patients		83	50	33
Age (years)		54 (19)	46 (19)	67 (11)
Gender (males/females)		67/16	36/14	31/2
NYHA	I	38 (46%)	36 (72%)	2 (6%)
	II	21 (25%)	11 (22%)	10 (30%)
	III	24 (29%)	3 (6%)	21 (63%)
**Echocardiographic characteristics**				
RWMS		21 (8)	16 (0)	30 (6)
AR ⩾3		13 (16%)	13 (26%)	0 (0%)
AS		7 (8%)	7 (14%)	0 (0%)
MR ⩾3		10 (12%)	10 (20%	0 (0%)
**cMR ejection fraction**		50 (16%)	59 (9%)	36 (14%)

AR, aortic regurgitation; AS, aortic stenosis; MR, mitral regurgitation; RWMS, regional wall motion score.

None of the patients were excluded because of poor image quality or non-interpretable 3-DE images. Thirteen patients had excellent (16%), 29 good (35%), 27 moderate (32%) and 14 patients fair (17%) 3-DE image quality. Manual corrections were necessary to optimise endocardial and epicardial contours in all patients for the two imaging methods. With 3-DE, these corrections resulted in only minor changes in the estimated epicardial LV volumes (on average 10 (15) ml from 282 (67) ml before correction to 292 (71) ml after correction). Correction also tended to be larger in patients with normal wall motion (NWM) (17 (10) ml) than in those with WMA (7 (16) ml).

Based on the 2-DE, 33 patients had wall motion abnormalities (WMA group, mean wall motion score: 30 (6)). Subjects with NWM (n = 50, NWM group) served as a controls.

### Comparison of 1-DE and cMR-derived LV mass

**Figure 2:**
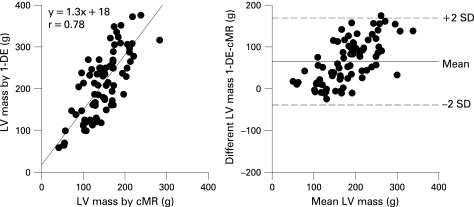
Linear regression and limits of agreement between estimates of LV mass by 1-DE and cMR.

Linear regression and Bland-Altman plots of LV mass by 1-DE versus cMR are shown in figure 2. 1-DE yielded significantly higher LV mass values than cMR (209 (77) g and 149 (42) g, respectively, p<0.001). Although both measurements correlated reasonably well (r = 0.78, p<0.001), a large bias was noted (62 (42) g).

### Comparison of 3-DE and cMR-derived LV mass

**Figure 3:**
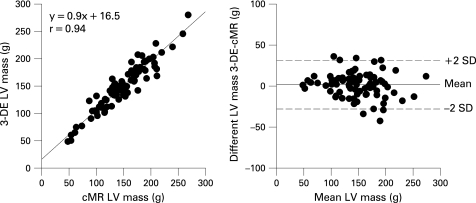
Linear regression and limits of agreement between estimates of LV mass by 3-DE and cMR.

Mean LV mass values by 3-DE and cMR are reported in table 2. Linear regression and Bland-Altman plots of LV mass by these two methods are shown in figure 3. LV mass by 3-DE and cMR were not significantly different from each other (149 (42) g and 148 (45) g, respectively, p = 0.67) and were highly correlated (r = 0.94, p<0.001).

**Table 2:** Mean values for LV mass, EDV, ESV, EF by 3-DE versus cMR

	3-DE	cMR	Comparison between 3-DE and cMR		
			r	Bias	p Value
LV mass (g)	149 (42)	148 (45)	0.94	1 (28)	0.67
EDV (ml)	167 (68)	187 (70)	0.97	−20 (31)	<0.001
ESV (ml)	88 (56)	101 (65)	0.98	−12 (31)	<0.001
EF (%)	50 (14)	50 (16)	0.94	1 (11)	0.23

EDV, end-diastolic volume; EF, ejection fraction; ESV, end-systolic volume; ICC, intraclass correlation coefficient.

### Measurement of LV mass in patients with and without wall motion abnormalities

**Figure 4:**
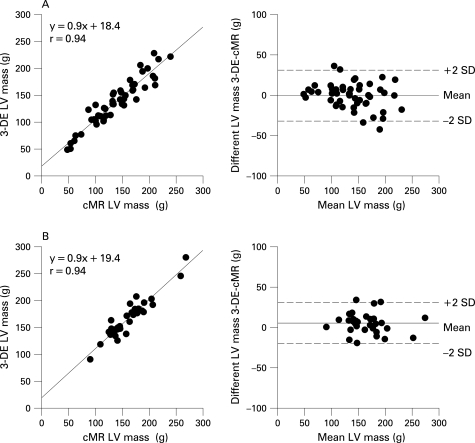
Linear regression and limits of agreement of estimates of LV mass in patients with normal wall motion (A) or with wall motion abnormalities (B) by 3-DE and cMR.

3-DE and cMR yielded similar values for LV mass in patients with (167 (35) g vs 164 (37) g, p = 0.11) and without (137 (43) g vs 138 (47) g, p = 0.77) WMA. In both groups, 3-DE and cMR values were highly correlated (r  =  0.94 each) with only small absolute biases (3 (20) g vs −1 932) g, p = 0.25) and relative biases (5% (8%) vs 9% (14%), p = 0.17) (fig 4).

### Comparison of LV volumes and ejection fraction between RT-3DE and cMR

**Figure 5:**
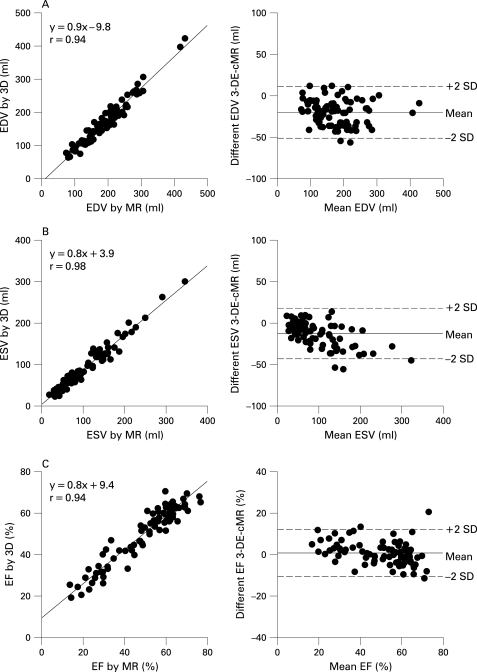
Linear regression and limits of agreement of estimates of end-diastolic volume (EDV) (A), end-systolic volume (ESV) (B) and ejection fraction (EF) (C) by 3-DE and cMR.

3-DE measurements of LV volumes and EF were also highly correlated with those obtained by cMR (r = 0.97, 0.98, 0.94 for EDV, ESV and EF, respectively). Yet, volumes were significantly underestimated by 3-DE (167 (68) ml vs 187 (70) ml for EDV, p<0.001; 88 (56) ml vs 101 (65) ml for ESV, p<0.001), resulting in small negative biases (−20 ml for EVD and −13 ml for ESV, both p<0.05, table 2 and fig 5A).

### Comparison of LV volumes and ejection fraction with and without injection of contrast

Use of contrast to opacify the LV cavity attenuated the differences between the two techniques (−3 ml for EDV and −3 ml for ESV, both p<0.05) but did not affect the measurement of EF (48% (15%) vs 50% (15%), p = 0.62). As shown in table 3, the extent to which contrast enhancement corrected the underestimation of LV volumes by 3-DE was similar in patients with (+20 ml for EDV and + 9 ml for ESV) and without (+18 ml for EDV and +3 ml for ESV) wall motion abnormalities (p = 0.68 and p = 0.08 for EDV and ESV respectively).

**Table 3:** Mean values for LV volumes measurements using unenhanced 3-DE, 3-D LVO and cMR in patients with RWMA (n = 11) and with NWM (n = 10)

		EDV (ml)	ESV (ml)	EF (%)
**3-DE**	**All groups**	**137 (46)**	**75 (47)**	**48 (15)**
	RWMA group	145 (55)	94 (56)	39 (14)
	NWM group	129 (33)	54 (20)	59 (4)
**3-D LVO**	**All groups**	**156 (49)**	**81 (51)**	**50 (15)**
	RWMA group	156 (59)	103 (61)	40 (15)
	NWM group	147 (35)	57 (20)	61 (6)
**cMR**	**All groups**	**159 (51)**	**84 (54)**	**50 (16)**
	RWMA group	168 (63)	107 (65)	39 (14)
	NWM group	149 (36)	58 (22)	59 (4)

NWM, normal wall motion; RWMA, regional wall motion abnormalities. Other abbreviations as in table 2.

### Reproducibility

Results of inter-observer and intra-observer reproducibility are summarised in table 4. The inter-observer and intra-observer reproducibility of 3-DE and cMR was similarly high. Test-retest reproducibility was also very good for both methods (table 5).

**Table 4:** Intra-observer and inter-observer reproducibility of LV mass measurements using 3-DE and cMR

		Reproducibility (ICC)		Limits of agreement Mean (2 SD)	
		Intra-observer	Inter-observer	Intra-observer	Inter-observer
RT-3DE	RWMA group	0.96	0.94	−3 (14)	3 (27)
	NWM group	0.97	0.94	−1 (26)	−6 (31)
MRI	RWMA group	0.94	0.95	−3 (18)	7 (26)
	NWM group	0.97	0.96	−1 (30)	2 (26)

ICC, intraclass correlation coefficient. Other abbreviations as in tables 2 and 3.

**Table 5:** Test-retest reproducibility

	Mean (SD) J0	Mean (SD) J1	Limits of agreement Mean (2 SD)	p Value
3-DE J0 vs J1	118 (25) g	121 (24) g	3 (9) g	0.22
cMR J0 vs J1	116 (35) g	116 (33) g	1 (8) g	0.88

## DISCUSSION

The results of our study indicate that 3-D imaging of the LV with either 3-DE or cMR produces comparable results in terms of LV volumes, EF and mass in patients with or without wall motion abnormalities. Our data also show that 3-DE and cMR provide similarly low inter-observer variability for all LV measurements and well as similarly high test-retest reproducibility.

### Accuracy of 3-DE assessment of LV volumes, ejection fraction and mass

Over the past 5 years, numerous studies have validated the use of 3-DE for assessment of LV volumes and EF, using various imaging methods as reference standards. All these studies have shown a greater accuracy of 3-DE compared with 1-DE or 2-DE.^9^^–^11 15,21^–^23 The results of this study confirm and extend these previous observations. As in previous studies, we found that semi-automated 3-DE measurements of LV volumes and EF were strongly correlated with those obtained by cMR. We also observed that 3-DE LV volumes were significantly underestimated compared with those obtained by cMR. Underestimation of LV volumes by 3-DE has been reported previously and has been attributed to technical limitations inherent in ultrasound imaging in general and 3-DE in particular, such as the inability to visualise the endocardial contours, the exclusion of trabecular structures from the LV cavity or the partial cut-off of the left ventricle.^24^ Divergent inclusion of the papillary muscles into the LV cavity between the two techniques has been incriminated as well. However, this was unlikely to be the case in our study, since the papillary muscles were treated in exactly the same way with both techniques. Interestingly, we found that the use of ultrasound contrast agents to opacify the LV cavity almost completely abolished the underestimation of LV volumes by 3-DE. This suggests that improved border delineation and exclusion of trabecular structures are the most likely contributors to the underestimation of LV volumes by 3-DE.

In the present study, we also evaluated the possibility of semi-automatically measuring LV mass using 3-DE. For this purpose, we modified the computerised contour-tracking algorithm used for endocardial border detection to allow for the estimation of the epicardial contours as well. Our data indicate that LV mass measurements obtained using this novel approach are highly accurate, even in patients whose LV cavity and walls are distorted by a previous myocardial infarction. So far, very few studies have examined the ability of 3-DE to measure LV mass. Although their results indicated that measurements of LV mass by 3-DE are feasible and correlate reasonably well with those obtained by cMR, most of these studies only recruited a limited number of patients (around 20 each), few investigated the accuracy of 3-DE in patients with distorted LV wall geometry^25^^–^27 and none used a semi-automated tracking algorithm that detects endocardial contours and facilitates epicardial contouring. To the best of our knowledge, the present study is the first to demonstrate the accuracy of such an approach in assessing LV mass in a large cohort of normal and diseased patients, with and without regional wall thickness abnormalities.

### Reproducibility of 3-DE assessment of LV volumes, ejection fraction and mass

Previous studies using 3-DE to measure LV volumes and EF have shown that this technique was more reproducible than 2-DE and as reproducible as cMR.^12^ ^22^ These favourable results probably reflect the combined use of 3-D datasets and semi-automated edge-detection algorithms, both of which contribute to better standardise the partition between the wall and cavity and hence to minimise variation. The results of our study are thus in line with these previous results. Our data also suggest that LV mass measurements from 3-DE images are as reproducible as those obtained by cMR, a particularly important finding for the follow-up of patients with LV hypertrophy or remodelling.

### Test-retest variation

In contrast with the more widely reported parameters of intra-observer and inter-observer variability, which relate to the repeated measurement of a single dataset, test-retest variation involves repetition of the entire acquisition and analysis. Assessment of test-retest variations is important because both physiological and imaging factors contribute to variations in LV function and mass over time. Previous work found significant test-retest variations with respect to both the angulation and displacement of 2-DE imaging planes. These variations partially explain the variability in 2-DE measurements of LV mass that have been highlighted in several studies. The present study demonstrates that test-retest variations in 3-DE and cMR measurements of LV volumes, EF and mass are quite similar. This is consistent with previous studies.^12^ ^20^ ^28^ This is an important finding because these measurements are often used to help make decisions about initiating or altering treatment in hypertensive subjects. They therefore need not only to be accurate but also reproducible over time. Since 3-DE is far more available and less costly than cMR, our findings suggest that 3-DE could become the ideal non-invasive imaging method to assess LV mass serially, whether in daily clinical practice or in the context of clinical trials on the regression of LV hypertrophy.

### Study limitations

First, the 3-D algorithm used to draw the epicardial contours is not yet fully automatic and still requires manual adjustments. Future development of the software should thus aim at tracking the epicardial contours automatically. Second, because of its robustness and reproducibility, we used cMR as the standard to which 3-DE measurements of LV volumes and mass were compared. When doing so, one should not forget that the assessment of LV parameters by cMR has its own limitations, including partial volume effects, the presence of a chemical shift artefact at the muscle-cavity boundary and uncertainties about whether or not to include the most basal slice of the cMR dataset into the measurements of LV volumes. Including or excluding the most basal cMR slice from the measurements can indeed result in a plus or minus 10% variation in estimated volumes.

## CONCLUSION

The results of our study indicate that 3-DE, in combination with semi-automated border detection, allows for a robust, accurate and reproducible quantification of LV volumes, EF and mass. The data also show that the robustness, accuracy and reproducibility of 3-DE is equal to that of cMR and similar in patients with and without regional wall motion abnormalities.
